# Correlation Theory of the Maxillary Central Incisor, Face and Dental Arch Shape in the Serbian Population

**DOI:** 10.3390/medicina59122142

**Published:** 2023-12-09

**Authors:** Milena Kostić, Nadica S. Đorđević, Nikola Gligorijević, Marija Jovanović, Ermin Đerlek, Kosta Todorović, Goran Jovanović, Jelena Todić, Marko Igić

**Affiliations:** 1 Clinic for Dental Medicine, Faculty of Medicine, University of Niš, 18000 Niš, Serbia; milena.kostic@medfak.ni.ac.rs (M.K.); nikola.gligorijevic@medfak.ni.ac.rs (N.G.); marija.jovanovic@medfak.ni.ac.rs (M.J.); kosta.todorovic@medfak.ni.ac.rs (K.T.); goran.jovanovic@medfak.ni.ac.rs (G.J.); marko.igic@medfak.ni.ac.rs (M.I.); 2 Department of Dentistry, Faculty of Medicine, University of Priština in Kosovska Mitrovica, 38220 Kosovska Mitrovica, Serbia; jelena.todic@med.pr.ac.rs; 3 Faculty of Medicine, University of Niš, 18000 Niš, Serbia; ermindjerlek@gmail.com

**Keywords:** maxillary central incisor, maxillary dental arch, face form, tooth form, Williams theory

## Abstract

*Background and Objectives*: According to the modified Williams theory, the shape of the maxillary central incisor corresponds to the shape of the maxillary alveolar ridge (dental arch) and the shape of the face. Moreover, the standards of beauty suggest that the center of the face of an individual with a full set of teeth should match the center of the maxillary and mandibular dental arches. The purpose of this study is to conduct a comparative cross-sectional study on the matching of the shape of the face, maxillary central incisor and maxillary dental arch as well as the matching of the midfacial line and dental arches in subjects with complete dentition. *Materials and Methods*: The study included 90 subjects of both sexes. The matching of the shape of the face and midfacial line, i.e., dental arches and maxillary incisors, was determined by analysing photographs, whereas the shape of the dental arch was determined by analysing plaster models. *Results*: No significant gender-related differences were found either in the shape of the maxillary central incisor that matched the shape of the maxillary dental arch (*p* = 0.349) or in the shape of the dental arch that matched the shape of the face (*p* = 0.697). However, a significant difference was noted in the shape of the teeth that matched the shape of the face (*p* = 0.043), which was more significantly impaired in men. In addition, the matching of the mid-face and the mid-dental arch was significantly greater in women (*p* = 0.016). *Conclusions*: The modified Williams theory was confirmed in most subjects, thus it can be considered a relevant guideline when determining the shape of teeth after their loss. The highest percentage of matching in both sexes was with the shape of the face and dental arch. There was no positive correlation between the middle of the maxillary and mandibular dental arch in most cases.

## 1. Introduction

The Williams theory of harmony or geometric theory was created in the first half of the twentieth century and represents an exact approach to determining the shape of teeth using anthropometric parameters that are available even after their loss [1]. It is different from the previously applied temperament theory, according to which the shape of teeth is matched based on a patient’s psychological constitution [2]. Leon Williams, a famous British dentist, observed the harmony of the shape of the maxillary central incisor and the outline of the face and divided the shape of teeth into oval, triangular, and square [3]. If the central incisor were enlarged and laid over the face so that the incisal edge is parallel to the eyebrows, and the neck of the tooth to the mandibular part of the face, then the shape of the tooth would match the shape of the face.

The Williams theory was based on an anthropometric study conducted on a thousand skulls at the Royal College of Georgia [4]. Even though Williams theory was not significantly scientifically supported at the time it was promoted, it undoubtedly became the simplest and most useful guide in choosing the shape of artificial teeth, especially after the classification was accepted by manufacturers.

In literature, the most common addition to the Williams theory is the finding by Boucher, who concluded that the shape and arrangement of dentures are also influenced by the shape of the alveolar ridge [5]. Therefore, it is expected that the shape of the face, maxillary central incisor, and alveolar ridge or dental arch of the maxillary jaw match [5]. This kind of modified Williams theory is found in textbooks for dental students as one of the guides for choosing artificial teeth in edentulous patients [5,6].

The matching of the midline of the face and dental arches is an essential part of prosthetic rehabilitation and orthodontic treatment. Regarding aesthetics, it is important to emphasize that the coordination of the midline of maxillary incisors with the midline of the face is more important than the coordination of the midline of mandibular incisors with the midline of the face, given that maxillary teeth are more visible during a pleasant smile [7]. For facial aesthetics and the choice of teeth for prosthetic rehabilitation, it is necessary to match the midline of the face and the upper dental arch. It is also desirable to match the middle of the upper and lower dental arches [8].

Although the Williams theory was introduced a long time ago and is now well-established, studies including the Serbian population have not yet been carried out. Conducting this study enables the comparison of the obtained results to recommendations for the clinical application of the theory.

The aim of the study is to conduct a comparative cross-sectional study on the matching of the shape of the face, maxillary central incisor and maxillary dental arch as well as the matching of the midfacial line and dental arches in subjects with full dentition.

## 2. Materials and Methods

The study was conducted at the Clinic for Dental Medicine in Niš and included 90 subjects. The subjects were dental students at the Faculty of Medicine in Niš. The study included only students with complete permanent dentition, with and without third molars, given that they had not erupted in all subjects by that time. Specifically, permanent dentition consists of 32 teeth, 16 maxillary and 16 mandibular teeth, i.e., 8 teeth on each side of the jaws. The evolution of permanent dentition begins around the age of 6 and ends at about 14–15 years of age when the roots have completed their development. The exception is related to third molars, which emerge at 18–25 years of age. The exclusion criteria were destruction of front teeth (caries, trauma, erosion), prosthetic and conservative restorations on front teeth, anomalies in the oral cavity, orthodontic therapy, congenital or surgical facial defects, or pronounced facial asymmetry.

To determine the sample size, we used a study by Khan and Kazmi [9] with 117 subjects, in which the proportion of maxillary and mandibular midlines amounted to 0.641. With a study precision criterion of 0.1 and confidence level of 95%, a sufficient number of observation units for the assessment of the proportion of patients with maxillary and mandibular midlines amounted to 88. Our study included 90 individuals. The subjects were familiar with the study and signed their written consent. The study was approved by the Ethics Committee of the Clinic for Dental Medicine (decision No. 14/7-2019-5EO).

**Figure 1:**
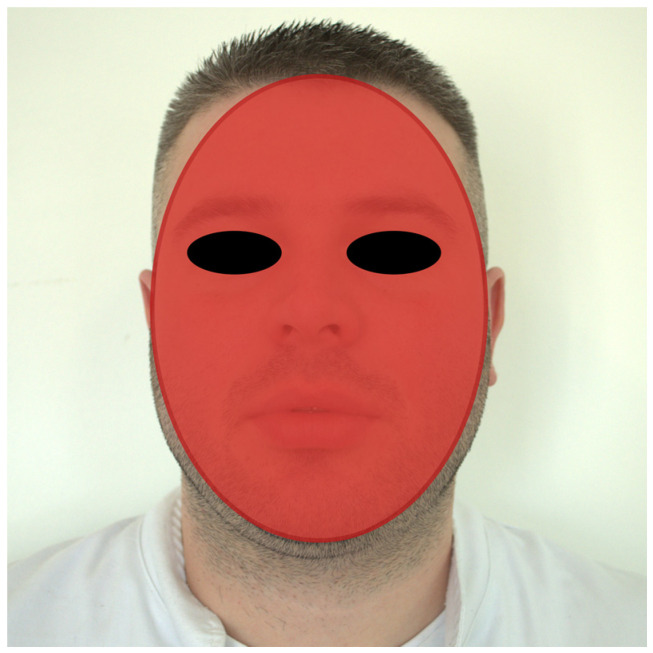
Outline tracing of the face.

The shape of the face was determined via digital analysis of the photographs of the subjects. The photography conditions were uniform for all subjects. The subjects were sitting with an upright position of body and head and looking towards the camera. The Frankfort plane and the bipupillary line were parallel to the axis of the camera objective that was positioned in the same plane for obtaining photographs [10]. The photos were taken with the lips joined together as well as with a smile to analyse the matching of the midline of the face and the middle of the dental arches. Standardized photographs were taken from a distance of 100 cm. The camera tripod (Olympus FE-200 digital camera (Olympus Corp., Tokyo, Japan)) was adjusted according to each subject. The hair did not cover any part of the face. Face images were then transferred to a personal computer with the Windows 11 operating system (Microsoft Corporation, Redmond, WA, USA) and digitally edited using image-editing software, Adobe Photoshop CC 2021 23.0 for Windows (Adobe Systems, San Jose, CA, USA). The facial outline form was determined from the outline of the temporal bone at the height of the trichion (tr) (the lowest point of the hairline in the centre of the face), the temporal process of the zygomatic arch, and the gonion (Figure 1) [11]. The face form was classified based on the Williams method [3,11] as follows:

(a) Square face form—the outline of the face between the temporal bone, zygomatic arch, and the gonion was parallel vertically;
(b) Tapering face form—the outline of the face from the temporal bone to the gonion was inwards vertically;
(c) Ovoid face form—the outline of the face from the temporal bone to the gonion was outwards vertically;

The analysis of the shape of the teeth and dental arch of each subject was performed after taking a maxillary alginate impression (Alginat Hydrogum 5, Zhermack S.p.A., Badia Polesine (RO), Italy) and casting a hard plaster model for analysis. The subjects were then photographed, and standardized photographs were taken from a distance of 40 cm where the camera was placed on a specially designed tripod, which enabled the uniformity of all photographs. The classification of tooth forms was also based on the Williams method [3,11,12]:

(a) Square incisor tooth,
(b) Ovoid incisor tooth,
(c) Tapering incisor tooth.

**Figure 2:**
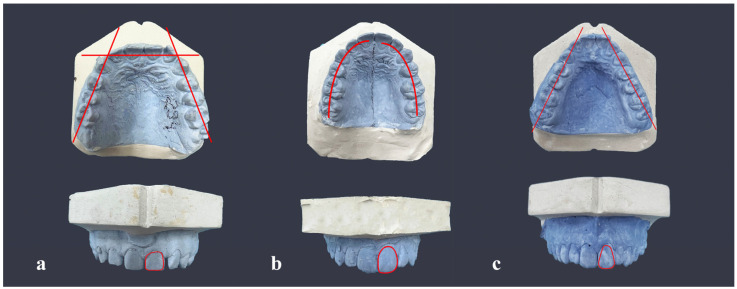
Matching of the shape of the maxillary dental arch and maxillary central incisor: (**a**) squared, (**b**) ovoid, (**c**) triangular.

The maxillary central incisor had the shape of a square if its proximal sides were parallel for at least half of the cervicoincisal length of the crown. In the case of a triangular incisor, the same surfaces converged cervically, whereas in the case of an oval incisor, they were biconvex (Figure 2). The analysis of the shape of the face, maxillary central incisor, and maxillary dental arch was undertaken by five dentists with at least eight years of work experience [11].

The matching of the midlines of the face and the maxillary dental arch and the midlines of the maxillary and the mandibular dental arch was conducted based on edited smile photographs that were analysed in the same program as that used for the face form and tooth forms.

The obtained data were statistically analyzed using descriptive statistical methods and methods for testing statistical hypotheses. The continuous variables are arithmetic mean, median, standard deviation, minimum (Min) and maximum (Max) value, whereas the categorical variables are absolute and relative numbers. For the continuous variable—age, the Shapiro-Wilk test was used to check the normality of the distribution. Given that age did not meet the normality of the distribution, a non-parametric method was used to compare the differences between two groups—the Mann-Whitney U test that tests the sum of ranks. The chi-square test was used to compare the differences in the frequency of the observed nominal variables between the sexes.

Statistical hypotheses were tested at the level of statistical significance α = 0.05. The software program SPSS Statistics 22 (SPSS Inc., Chicago, IL, USA) was used for statistical processing of the results.

## 3. Results

The study included 90 subjects, aged 20 to 27. The mean age was 22.2 ± 1.5. Out of the total number of patients, 32 (35.6%) were male and 58 (64.4%) were female.

The mean age of male and female patients was 22 (range 20–25) and 22 (range 20–27), respectively. The ages of the subjects did not statistically significantly differ regarding gender (U = 903.5, *p* = 0.832).

The correlation of the shape of the central maxillary incisor, maxillary dental arch, and face of all subjects as well as the differences between the sexes are shown in Table 1. A positive correlation among the shapes was observed in all three segments of the examination. The correlation among the shapes of the central maxillary incisor, maxillary dental arch and face of all subjects as well as the differences between the sexes are shown in Table 1. A positive correlation among the shapes was observed in all three segments of the examination.

Based on gender, no statistically significant difference was found in the shape of teeth corresponding to the shape of the dental arch (χ^2^ = 0.878; *p* = 0.349) and the shape of the dental arch corresponding to the shape of the face (χ^2^ = 0.152; *p* = 0.697). However, there was a statistically significant difference in the shape of teeth corresponding to the shape of the face (χ^2^ = 4.097; *p* = 0.043), and percentagewise it was less represented in women.

In most subjects, the middle of the face matched the middle of the maxillary dental arch. The matching of the middle of the face and the middle of the dental arch differed significantly between men and women (χ^2^ = 5.757; *p* = 0.016). A statistically significantly greater matching was observed in women.

The midline of the maxillary and mandibular dental arches was not uniform in most subjects. There was no significance in the matching of the middle of the maxillary and mandibular dental arch based on gender (χ^2^ = 1.968; *p* = 0.161) (Table 2).

## 4. Discussion

The Williams theory is based on the concordance of the form of the inverted and enlarged maxillary central incisor and the outline of the face and applies to all races [2]. The modification of the Williams theory implies comparing the shape of the face and the maxillary central incisor with the shape of the dental arch, which provides relevant data in determining the shape of teeth after their loss. The purpose of this analysis is to restore the aesthetics and function of the orofacial system in patients, especially those who lost their front or all teeth in the jaws, i.e., to create a smile that restores their natural appearance and self-confidence in social contact.

Previous tests of the credibility of the Williams theory have been carried out using the visual method or digital analysis of the photographs of subjects [13]. The advantages of using photographs to analyse the shape of the face are reflected in easier observation and comparison after software processing. Given that the Williams theory and its modified form have long been present in the literature and used in practice, computer processing provides a new dimension to this method of tooth shape selection, making it more precise, repeatable and available.

The study included 90 dental students of both sexes with complete dentition in both jaws. Subjects with orthodontic anomalies and hard and soft oral structure diseases were excluded from the study. The homogenous group ensured the relevance of data, on which recommendations for the application of the modified Williams theory on the Serbian population can be based. In the analysis of the shape of the face, photos with closed competent lips and no smile were used. The shape of the maxillary central incisor and maxillary dental arch were analysed using work models [1].

The obtained results justified the use of the modified Williams theory in dental practice, given that the positive correlations of the shape of the central maxillary incisor and the face, the central maxillary incisor and the maxillary dental arch, and the maxillary dental arch and the face were confirmed. However, in female subjects, the percentage of matching of the shape of the face and teeth was mandibular. A higher positive correlation of the studied forms was reported in male subjects.

Analogous to the results of this study, studies by Shaweesh et al. and Agarwal et al. showed that there is a connection between the shape of the face and the maxillary central incisors [14,15]. The most common was the combination of an oval face and oval incisors (in 76% of cases in men and 61% of cases in women). A combination of square shapes was reported in 11% of male and 14% of female subjects, that is, the combination of triangular shapes in 6% of men and 12% of women [15]. Habib et al. suggested that square is the most common shape of the face (49.65%) and incisors (56.38%). Using the visual method, the observers found a good relationship (31.41%), a moderate relationship (35.31%), a weak relationship (19.68%), and no relationship (13.65%) between the shape of teeth and the shape of the face [16].

Sellen et al. compared the shape of the teeth, face, dental arch, and palate in 50 subjects using plaster models and photographs. The shape of the face and the central incisor matched in 22% of the cases, which was the lowest match. The shape of the palate and the dental arch matched in 44% of the cases, which was the highest. The shape of the face and the dental arch matched in 28% of the subjects, and the shape of the maxillary incisor and the maxillary dental arch matched in 24% of the subjects [17]. Studies by numerous authors have not supported the Williams theory [18,19]. Brisman criticized it by proving with a photometric study on 80 subjects that the matching degree of the shape of the central maxillary incisors and the face is extremely small. In addition, the same author does not consider the matching of the described values necessarily beautiful, referring to a different perception of aesthetics by dentists and patients [20]. Wright found that only 30% of subjects had a match between the shape of teeth and the face [21]. Varjão et al. showed that there is no highly defined correlation between the shape of the central incisor and the shape of the face in any of the studied racial groups [22].

Koralakunte and Budilah criticized the Williams theory stating that the shape discrepancy does not exist due to the frequent asymmetry of the maxillary incisor against the normally symmetrical face [11]. Considering that artificial teeth can only partially copy the variety of details in nature, this theory serves as reasonable compensation to achieve a good aesthetic effect.

Seluk et al. studied the possibility of choosing the shape of teeth using the shape of the face by making each subject, from a group of six edentulous patients (three men and three women), three different prostheses with round, square, and oval teeth. The patient’s preference for a certain tooth shape was not in accordance with the Williams theory [23]. Regardless of the debate about the accuracy and aesthetic effect after the application of the tested theory, the modelling of the gingiva around the artificial teeth significantly influenced the change in the shape of the teeth from that of the dental sets, as shown by studies of numerous authors [24].

Empirical data certainly indicate the applicability of the Williams theory in everyday clinical practice. It can unquestionably be supplemented by the SPA theory (sex, personality, age), whereby the choice of teeth would also be determined by gender (relation between central and lateral incisors, roundness of tooth lines), personality and age of the patient (colour and abrasion) [25].

The second part of the study referred to the matching of the midline of the face and the midline of the dental arches. Photographs of the subjects with smiles were used to observe the midlines of the upper and lower dental arches. The height of the upper lip and the dynamics of the smile were not taken into account as variables in the perception of a patient’s appearance in relation to the matching of the midlines.

The results of this study showed that in most subjects (69.2%), there was a match between the middle of the face and the middle of the maxillary dental arch, especially in women. The described match is one of the important parameters of beauty, given the perceptibility in everyday contact with the subject. The obtained results are comparable with the results of other authors [10,26].

The congruence of the medial line of the face and the medial line of the maxillary dental arch is considered one of the aesthetic imperatives and must be respected in dental prosthetic rehabilitation. A deviation of 1 to 2 mm cannot be detected by the eye [8,27,28].

The middle of the maxillary and mandibular dental arch did not match in most subjects. This discrepancy was percentagewise higher in men. According to literature data, the mismatch of the maxillary and mandibular dental arch is significantly more common than the mismatch of the midline of the maxillary dental arch and the midline of the face [29]. On the other hand, it is difficult to notice and does not significantly disturb the appearance of the subject. It is the result of certain orthodontic anomalies or more frequently, unilateral tooth loss. In contrast, Khan and Kazmi described frequent matching of the midlines of dental arches but not their matching with the midface [9]. If it is impossible to achieve the matching of these two lines with therapy, the line between the central incisors must be parallel to the central line of the face [25].

The aesthetics of prosthetic works is as important as their functionality in the orofacial system. After the loss of front or all teeth in the upper or lower jaw, adequate occlusal rehabilitation is necessary. Among other things, it implies the correct selection of teeth, the most important of which is the selection of the maxillary incisor, which is the most visible when speaking and smiling. One of the guidelines for its detection can be the long-used Williams theory with its modifications, especially in the era of software use that has the possibility of designing smiles. The matching of the midline of the face and the midline of the maxillary central incisors is an essential parameter of the overall appearance of the face and must be respected in prosthetic and restorative rehabilitation. On the other hand, the matching of the midline of the maxillary and mandibular dental arches is not frequent, and not necessary whatsoever, but it should be emphasized in all clinical cases where possible.

## 5. Conclusions

The highest percentage match refers to the shape of the face and dental arch in both sexes. The modified Williams theory is not exact, but the results obtained in patients with a full set of teeth showed its usability in a larger number of cases. This qualifies it as one of the methods for tooth selection in edentulous patients. The modelling of the gingiva and the adequate setting of the teeth give a subjective tone to prosthetic restoration. A patient’s perception of aesthetics needs to be considered when making all types of prosthetic restorations, given that it is what gives each of them individuality.

There was no positive correlation between the middle of the maxillary and mandibular dental arch in most cases. From an aesthetic point of view, matching the midline of the maxillary dental arch with the midline of the face is essential.

The use of new technologies and the analysis of results using software has certainly improved the relevance of the obtained conclusions, based on which a foundation for the selection of teeth after tooth loss can be obtained.

## Figures and Tables

**Table 1:** Correlation among the shapes of the maxillary incisor, maxillary dental arch, and face.

	Total		Men		Women		*p*
	n	(%)	n	(%)	n	(%)	
The shape of teeth corresponds to the facial shape							
no	41	45.6	10	31.2	31	53.4	
yes	49	54.4	22	68.8	27	46.6	0.043 *
The shape of teeth corresponds to the dental arch shape							
no	31	34.4	9	28.1	22	37.9	
yes	59	65.6	23	71.9	36	62.1	0.349
The shape of the dental arch corresponds to the facial shape							
no	10	11.1	3	9.4	7	12.1	
yes	80	88.9	29	90.6	51	87.9	0.697

Data were tested using the χ^2^ test. n-number of subjects, * *p*-value < 0.05 = statistically significant difference between gender.

**Table 2:** Correlations between the middle of the face and the middle of the dental arch and the middle of the maxillary and mandibular dental arch based on gender.

		Total		Men		Women		*p* *
		n	%	n	%	n	%	
The match of the middle of the face and the middle of the dental arch	no	28	31.1	15	46.9	13	22.4	0.016 *
	yes	62	68.9	17	53.1	45	77.6	
The match of the middle of the maxillary and mandibular dental arch	no	56	62.2	23	71.9	33	56.9	0.161
	yes	34	37.8	9	28.1	25	43.1	

Data were tested using the χ^2^ test. n-number of subjects, * *p*-value < 0.05 = statistically significant difference between gender

## Data Availability

The data presented in this study are available on request from the corresponding author.
